# Supplementary material for: Asymptotic standard errors for reliability coefficients in item response theory
Source: Br J Math Stat Psychol. 2026 Mar 26;79(3):702–19. doi: 10.1111/bmsp.70047 (PMC13679761; doi:10.1111/bmsp.70047)
Supplement: Supplementary file 1 [file BMSP-79-702-s001.zip › Supplementary Document.pdf]

Supplementary Document for
"Asymptotic Standard Errors for Reliability Coefficients in Item Response Theory"

Youjin Sung[1] and Yang Liu

Contents

A Derivations of Asymptotic Standard Errors **1**
- A.1 General Theory 1
- A.2 PRMSE for the LV 2
- A.3 CTT Reliability for the EAP Score 4

B True Item Parameters **6**
- B.1 2PL Model 6
- B.2 Graded Response Model 7

C Additional Simulation Results **9**
- C.1 Simulation Setup 9
- C.2 Results 9

D Normalized CTT Reliability **14**
- D.1 Normalized Formula 14
- D.2 Additional Derivations 16
- D.3 Simulation Results 17

[1]University of Maryland, College Park. Correspondence should be made to Youjin Sung at 1232 Benjamin Bldg, 3942 Campus Dr, University of Maryland, College Park, MD, 20742. Email: yjsung@umd.edu.

A Derivations of Asymptotic Standard Errors

A.1 General Theory

To derive the asymptotic standard error (SE) of $\varphi(\hat{\boldsymbol{\eta}}(\hat{\boldsymbol{\nu}}))$, we first study the asymptotic behavior of $\hat{\boldsymbol{\eta}}(\hat{\boldsymbol{\nu}})$ before any transformation. Rewrite

$$
\begin{aligned}
&\sqrt{n}\left[\hat{\boldsymbol{\eta}}(\hat{\boldsymbol{\nu}}) - \boldsymbol{\eta}(\boldsymbol{\nu})\right] \\
&= \sqrt{n}\left[\hat{\boldsymbol{\eta}}(\hat{\boldsymbol{\nu}}) - \hat{\boldsymbol{\eta}}(\boldsymbol{\nu})\right] + \sqrt{n}\left[\hat{\boldsymbol{\eta}}(\boldsymbol{\nu}) - \boldsymbol{\eta}(\boldsymbol{\nu})\right]. \qquad \text{(S1)}
\end{aligned}
$$

Let $p_n(\mathbf{y}_i)$ and $f(\mathbf{y}_i;\boldsymbol{\nu})$ be the sample proportion and model-implied probability of response pattern $\mathbf{y}_i$. Using these notations, the first term on the right-hand side of Equation S1 is expressed as

$$
\begin{aligned}
&\sqrt{n}\left[\hat{\boldsymbol{\eta}}(\hat{\boldsymbol{\nu}}) - \hat{\boldsymbol{\eta}}(\boldsymbol{\nu})\right] \\
&= \sqrt{n}\sum_{\mathbf{y}_i} p_n(\mathbf{y}_i)\left[\mathbf{H}(\mathbf{y}_i;\hat{\boldsymbol{\nu}}) - \mathbf{H}(\mathbf{y}_i;\boldsymbol{\nu})\right] \\
&= \underbrace{\sqrt{n}\sum_{\mathbf{y}_i}\left[p_n(\mathbf{y}_i) - f(\mathbf{y}_i;\boldsymbol{\nu})\right]\left[\mathbf{H}(\mathbf{y}_i;\hat{\boldsymbol{\nu}}) - \mathbf{H}(\mathbf{y}_i;\boldsymbol{\nu})\right]}_{\text{(I)}} \\
&\quad + \underbrace{\sqrt{n}\sum_{\mathbf{y}_i} f(\mathbf{y}_i;\boldsymbol{\nu})\left[\mathbf{H}(\mathbf{y}_i;\hat{\boldsymbol{\nu}}) - \mathbf{H}(\mathbf{y}_i;\boldsymbol{\nu})\right]}_{\text{(II)}}. \qquad \text{(S2)}
\end{aligned}
$$

(I) in Equation S2 is $o_p(1)$ because (a) for each $\mathbf{y}_i$, $\sqrt{n}[p_n(\mathbf{y}_i) - f(\mathbf{y}_i;\boldsymbol{\nu})] = O_p(1)$ as $n \to \infty$ by the asymptotic normality of sample proportions, (b) $\hat{\boldsymbol{\nu}} \xrightarrow{p} \boldsymbol{\nu}$ as $n \to \infty$ by the consistency of the maximum likelihood (ML) estimator, and (c) $\mathbf{H}$ is continuous in $\boldsymbol{\nu}$. Now applying the Delta method to (II) gives

$$
\begin{aligned}
\text{(II)} &= \sum_{\mathbf{y}_i} f(\mathbf{y}_i;\boldsymbol{\nu})\nabla_{\boldsymbol{\nu}}\mathbf{H}(\mathbf{y}_i;\boldsymbol{\nu})\sqrt{n}(\hat{\boldsymbol{\nu}} - \boldsymbol{\nu}) \\
&= \mathbb{E}\left[\nabla_{\boldsymbol{\nu}}\mathbf{H}(\mathbf{Y}_i;\boldsymbol{\nu})\right]^{\top}\boldsymbol{\mathcal{I}}^{-1}(\boldsymbol{\nu})\sqrt{n}\nabla_{\boldsymbol{\nu}}\hat{\ell}(\boldsymbol{\nu}) + o_p(1), \qquad \text{(S3)}
\end{aligned}
$$

in which $\nabla_{\boldsymbol{\nu}}\mathbf{H}$ produces a $Km \times \ell$ gradient matrix for $\mathbf{H} \in \mathbb{R}^{\ell}$, and $\nabla_{\boldsymbol{\nu}}\hat{\ell}(\boldsymbol{\nu}) = n^{-1}\sum_{i=1}^{n}\nabla_{\boldsymbol{\nu}}\log f(\mathbf{Y}_i;\boldsymbol{\nu})$ from Equation 8 of the main document. Combining

Equations S2 and S3 yields a further expression of Equation S1:

$$\begin{aligned}
&\sqrt{n}\left[\hat{\boldsymbol{\eta}}(\hat{\boldsymbol{\nu}})-\boldsymbol{\eta}(\boldsymbol{\nu})\right]\\
=&\left(\mathbb{E}[\nabla_{\boldsymbol{\nu}}\mathbf{H}(\mathbf{Y}_i;\boldsymbol{\nu})]^{\top}\boldsymbol{\mathcal{I}}^{-1}(\boldsymbol{\nu}):\mathbf{I}_{\ell\times\ell}\right)\sqrt{n}\begin{pmatrix}\nabla_{\boldsymbol{\nu}}\hat{\ell}(\boldsymbol{\nu})\\ \hat{\boldsymbol{\eta}}(\boldsymbol{\nu})-\boldsymbol{\eta}(\boldsymbol{\nu})\end{pmatrix}+o_p(1)\\
\xrightarrow{d}&\,\mathcal{N}\left(\mathbf{0},\boldsymbol{\Sigma}(\boldsymbol{\nu})\right),
\end{aligned}\tag{S4}$$

in which

$$\boldsymbol{\Sigma}(\boldsymbol{\nu})=\left(\mathbb{E}[\nabla_{\boldsymbol{\nu}}\mathbf{H}(\mathbf{Y}_i;\boldsymbol{\nu})]^{\top}\boldsymbol{\mathcal{I}}^{-1}(\boldsymbol{\nu}):\mathbf{I}_{\ell\times\ell}\right)\boldsymbol{\Omega}(\boldsymbol{\nu})\left(\mathbb{E}[\nabla_{\boldsymbol{\nu}}\mathbf{H}(\mathbf{Y}_i;\boldsymbol{\nu})]^{\top}\boldsymbol{\mathcal{I}}^{-1}(\boldsymbol{\nu}):\mathbf{I}_{\ell\times\ell}\right)^{\top}.\tag{S5}$$

Note that the colon (:) in the second line of Equation S4 denotes column-wise concatenation of matrix blocks. Additionally, $\boldsymbol{\Omega}(\boldsymbol{\nu})$ in Equation S5 denotes the covariance matrix of the random vector $[\nabla_{\boldsymbol{\nu}}\log f(\mathbf{Y}_i;\boldsymbol{\nu})^{\top}:\mathbf{H}(\mathbf{Y}_i;\boldsymbol{\nu})^{\top}]^{\top}$:

$$\boldsymbol{\Omega}(\boldsymbol{\nu})=\begin{pmatrix}\boldsymbol{\mathcal{I}}(\boldsymbol{\nu}) & \mathbf{A}(\boldsymbol{\nu})\\ \mathbf{A}(\boldsymbol{\nu})^{\top} & \boldsymbol{\Sigma}_{\mathbf{H}}(\boldsymbol{\nu})\end{pmatrix},\tag{S6}$$

in which $\mathbf{A}(\boldsymbol{\nu})=\mathrm{Cov}\left[\mathbf{H}(\mathbf{Y}_i;\boldsymbol{\nu}),\nabla_{\boldsymbol{\nu}}\log f(\mathbf{Y}_i;\boldsymbol{\nu})^{\top}\right]$ and $\boldsymbol{\Sigma}_{\mathbf{H}}(\boldsymbol{\nu})=\mathrm{Cov}[\mathbf{H}(\mathbf{Y}_i;\boldsymbol{\nu})]$.

Now, apply the Delta method to obtain the asymptotic distribution of the reliability coefficient $\varphi(\hat{\boldsymbol{\eta}}(\hat{\boldsymbol{\nu}}))$:

$$\begin{aligned}
\sqrt{n}\left[\varphi(\hat{\boldsymbol{\eta}}(\hat{\boldsymbol{\nu}}))-\varphi(\boldsymbol{\eta}(\boldsymbol{\nu}))\right]&=\sqrt{n}\nabla\varphi\left(\boldsymbol{\eta}(\boldsymbol{\nu})\right)^{\top}\left[\hat{\boldsymbol{\eta}}(\hat{\boldsymbol{\nu}})-\boldsymbol{\eta}(\boldsymbol{\nu})\right]+o_p(1)\\
&\xrightarrow{d}\mathcal{N}\left(0,\nabla\varphi(\boldsymbol{\eta}(\boldsymbol{\nu}))^{\top}\boldsymbol{\Sigma}(\boldsymbol{\nu})\nabla\varphi(\boldsymbol{\eta}(\boldsymbol{\nu}))\right).
\end{aligned}\tag{S7}$$

Equation S7 is the final expression, identical to Equation 22 in the main document. In the following subsections, we provide the expressions for $\nabla_{\boldsymbol{\nu}}\mathbf{H}(\mathbf{Y}_i;\boldsymbol{\nu})$ and $\nabla\varphi(\boldsymbol{\eta}(\boldsymbol{\nu}))$, derived specifically for the two reliability coefficients: PRMSE for the latent variable (LV) and CTT reliability for the expected *a posteriori* (EAP) score.

A.2 PRMSE for the LV

For the PRMSE coefficient, we defined the function $\mathbf{H}$ in Equation 25 as

$$\mathbf{H}(\mathbf{Y}_i;\boldsymbol{\nu})=(H_1(\mathbf{Y}_i;\boldsymbol{\nu}),H_2(\mathbf{Y}_i;\boldsymbol{\nu}),H_3(\mathbf{Y}_i;\boldsymbol{\nu}))^{\top},\tag{S8}$$

in which $H_1(\mathbf{Y}_i;\boldsymbol{\nu})=\mathbb{E}(\Theta_i|\mathbf{Y}_i;\boldsymbol{\nu})$, $H_2(\mathbf{Y}_i;\boldsymbol{\nu})=\mathbb{E}(\Theta_i|\mathbf{Y}_i;\boldsymbol{\nu})^2$, and $H_3(\mathbf{Y}_i;\boldsymbol{\nu})=\mathrm{Var}(\Theta_i|\mathbf{Y}_i;\boldsymbol{\nu})$. The gradients of functions H_1, H_2, and H_3 with respect to $\boldsymbol{\nu}$ are derived as follows:

$$
\begin{aligned}
\nabla_{\boldsymbol{\nu}} H_1(\mathbf{Y}_i;\boldsymbol{\nu}) = \nabla_{\boldsymbol{\nu}} \mathbb{E}(\Theta_i|\mathbf{Y}_i;\boldsymbol{\nu}) &= \nabla_{\boldsymbol{\nu}} \int \theta_i f(\theta_i|\mathbf{y}_i;\boldsymbol{\nu}) d\theta_i \\
&= \int \theta_i \phi(\theta_i) \nabla_{\boldsymbol{\nu}} \left[\frac{f(\mathbf{y}_i|\theta_i;\boldsymbol{\nu})}{f(\mathbf{y}_i;\boldsymbol{\nu})} \right] d\theta_i,
\end{aligned} \tag{S9}
$$

$$
\nabla_{\boldsymbol{\nu}} H_2(\mathbf{Y}_i;\boldsymbol{\nu}) = \nabla_{\boldsymbol{\nu}} \mathbb{E}(\Theta_i|\mathbf{Y}_i;\boldsymbol{\nu})^2 = 2H_1(\mathbf{Y}_i;\boldsymbol{\nu}) \nabla_{\boldsymbol{\nu}} H_1(\mathbf{Y}_i;\boldsymbol{\nu}), \tag{S10}
$$

and

$$
\begin{aligned}
\nabla_{\boldsymbol{\nu}} H_3(\mathbf{Y}_i;\boldsymbol{\nu}) = \nabla_{\boldsymbol{\nu}} \mathrm{Var}(\Theta_i|\mathbf{Y}_i;\boldsymbol{\nu}) &= \nabla_{\boldsymbol{\nu}} \mathbb{E}(\Theta_i^2|\mathbf{Y}_i;\boldsymbol{\nu}) - \nabla_{\boldsymbol{\nu}} \mathbb{E}(\Theta_i|\mathbf{Y}_i;\boldsymbol{\nu})^2 \\
&= \int \theta_i^2 \phi(\theta_i) \nabla_{\boldsymbol{\nu}} \left[\frac{f(\mathbf{y}_i|\theta_i;\boldsymbol{\nu})}{f(\mathbf{y}_i;\boldsymbol{\nu})} \right] d\theta_i - \nabla_{\boldsymbol{\nu}} H_2(\mathbf{Y}_i;\boldsymbol{\nu}),
\end{aligned} \tag{S11}
$$

in which the integrals are approximated using quadrature. In Equations S9–S11, the gradient of the bracketed term can be rewritten as:

$$
\begin{aligned}
&\nabla_{\boldsymbol{\nu}} \left[\frac{f(\mathbf{y}_i|\theta_i;\boldsymbol{\nu})}{f(\mathbf{y}_i;\boldsymbol{\nu})} \right] \\
&= \frac{f(\mathbf{y}_i|\theta_i;\boldsymbol{\nu})}{f(\mathbf{y}_i;\boldsymbol{\nu})} \left[\nabla_{\boldsymbol{\nu}} \log f(\mathbf{y}_i|\theta_i;\boldsymbol{\nu}) - \nabla_{\boldsymbol{\nu}} \log f(\mathbf{y}_i;\boldsymbol{\nu}) \right] \\
&= \frac{f(\mathbf{y}_i|\theta_i;\boldsymbol{\nu})}{f(\mathbf{y}_i;\boldsymbol{\nu})} \left[\nabla_{\boldsymbol{\nu}} \log f(\mathbf{y}_i|\theta_i;\boldsymbol{\nu}) - \frac{\int \nabla_{\boldsymbol{\nu}} \log f(\mathbf{y}_i|\theta_i;\boldsymbol{\nu}) f(\mathbf{y}_i|\theta_i;\boldsymbol{\nu}) \phi(\theta_i) d\theta_i}{f(\mathbf{y}_i;\boldsymbol{\nu})} \right].
\end{aligned} \tag{S12}
$$

Recall that under the graded response model (GRM) with K response categories, $\log f(\mathbf{y}_i|\theta_i;\boldsymbol{\nu}) = \sum_{j=1}^{m} \log f_j(y_{ij}|\theta_i; a_j, c_{j1}, \ldots, c_{j,K-1})$, where the item response function f_j is obtained by the difference in f_j^* functions with adjacent categories

$$
f_j(k|\theta_i;\boldsymbol{\nu}) = f_j^*(k|\theta_i;\boldsymbol{\nu}) - f_j^*(k+1|\theta_i;\boldsymbol{\nu}), \quad k = 0, \ldots, K-1, \tag{S13}
$$

in which $f_j^*(k|\theta_i;\boldsymbol{\nu}) = \mathbb{P}\{Y_{ij} \geq k|\theta_i;\boldsymbol{\nu}\}$ is parameterized by the two-parameter logistic (2PL) model (see Equation 3 in the main document). For each item j, we express the derivative of the log item response function $\log f_j$ with respect to a_j as follows:

$$
\begin{aligned}
&\frac{\partial}{\partial a_j} \log f_j(y_{ij} = k|\theta_i; a_j, c_{j1}, \ldots, c_{j,K-1}) \\
&= \frac{\frac{\partial}{\partial a_j}(f_j^*(k|\theta_i;\boldsymbol{\nu}) - f_j^*(k+1|\theta_i;\boldsymbol{\nu}))}{f_j(k|\theta_i;\boldsymbol{\nu})} \\
&= \frac{f_j^*(k|\theta_i;\boldsymbol{\nu}) \frac{\partial}{\partial a_j} \log f_j^*(k|\theta_i;\boldsymbol{\nu}) - f_j^*(k+1|\theta_i;\boldsymbol{\nu}) \frac{\partial}{\partial a_j} \log f_j^*(k+1|\theta_i;\boldsymbol{\nu})}{f_j(k|\theta_i;\boldsymbol{\nu})},
\end{aligned} \tag{S14}
$$

in which

$$\frac{\partial}{\partial a_j} \log f_j^*(k|\theta_i;\boldsymbol{\nu}) = \theta_i \left[1 - f_j^*(k|\theta_i;\boldsymbol{\nu})\right]. \tag{S15}$$

Similarly, the derivative with respect to c_{jk} is

$$\begin{aligned}
&\frac{\partial}{\partial c_{jk}} \log f_j(y_{ij} = k|\theta_i; a_j, c_{j1}, \ldots, c_{j,K-1}) \\
&= \frac{\frac{\partial}{\partial c_{jk}}(f_j^*(k|\theta_i;\boldsymbol{\nu}) - f_j^*(k+1|\theta_i;\boldsymbol{\nu}))}{f_j(k|\theta_i;\boldsymbol{\nu})} \\
&= \frac{f_j^*(k|\theta_i;\boldsymbol{\nu})\frac{\partial}{\partial c_{jk}}\log f_j^*(k|\theta_i;\boldsymbol{\nu}) - f_j^*(k+1|\theta_i;\boldsymbol{\nu})\frac{\partial}{\partial c_{jk}}\log f_j^*(k+1|\theta_i;\boldsymbol{\nu})}{f_j(k|\theta_i;\boldsymbol{\nu})},
\end{aligned} \tag{S16}$$

in which

$$\frac{\partial}{\partial c_{jk}} \log f_j^*(k|\theta_i;\boldsymbol{\nu}) = 1 - f_j^*(k|\theta_i;\boldsymbol{\nu}). \tag{S17}$$

To compute PRMSE for the latent variable, we apply the transformation function $\varphi_{\text{PRMSE}}(\mathbf{x}) = (x_2 - x_1^2)/(x_2 - x_1^2 + x_3)$ where $\mathbf{x} = (x_1, x_2, x_3)^\top$. The gradient of φ_{PRMSE} is

$$\nabla\varphi_{\text{PRMSE}}(\mathbf{x}) = \left(\frac{-2x_1x_3}{(x_2 - x_1^2 + x_3)^2}, \frac{x_3}{(x_2 - x_1^2 + x_3)^2}, \frac{x_1^2 - x_2}{(x_2 - x_1^2 + x_3)^2}\right)^\top. \tag{S18}$$

A.3 CTT Reliability for the EAP Score

For the CTT reliability coefficient, we defined $\mathbf{H}$ in Equation 33 as

$$\mathbf{H}(\mathbf{Y}_i;\boldsymbol{\nu}) = (H_1(\mathbf{Y}_i;\boldsymbol{\nu}), H_2(\mathbf{Y}_i;\boldsymbol{\nu}), H_3(\mathbf{Y}_i;\boldsymbol{\nu}), \ldots, H_{2+Q}(\mathbf{Y}_i;\boldsymbol{\nu}))^\top, \tag{S19}$$

in which $H_1(\mathbf{Y}_i;\boldsymbol{\nu})$ and $H_2(\mathbf{Y}_i;\boldsymbol{\nu})$ are the same as in PRMSE, and the remaining functions are defined as

$$H_{2+q}(\mathbf{Y}_i;\boldsymbol{\nu}) = H_1(\mathbf{Y}_i;\boldsymbol{\nu})\frac{f(\mathbf{Y}_i|\theta_{iq};\boldsymbol{\nu})}{f(\mathbf{Y}_i;\boldsymbol{\nu})},\ q = 1, \ldots, Q. \tag{S20}$$

Here, θ_{iq} denotes the q-th quadrature point used to approximate the integration with respect to the LV density, and Q denotes the total number of the quadrature points. The gradient of Equation S20 is expressed by

$$\begin{aligned}
\nabla_{\boldsymbol{\nu}} H_{2+q}(\mathbf{Y}_i;\boldsymbol{\nu}) &= \nabla_{\boldsymbol{\nu}}\left[H_1(\mathbf{Y}_i;\boldsymbol{\nu})\frac{f(\mathbf{Y}_i|\theta_{iq};\boldsymbol{\nu})}{f(\mathbf{Y}_i;\boldsymbol{\nu})}\right] \\
&= \nabla_{\boldsymbol{\nu}} H_1(\mathbf{Y}_i;\boldsymbol{\nu})\frac{f(\mathbf{Y}_i|\theta_{iq};\boldsymbol{\nu})}{f(\mathbf{Y}_i;\boldsymbol{\nu})} + H_1(\mathbf{Y}_i;\boldsymbol{\nu})\nabla_{\boldsymbol{\nu}}\left[\frac{f(\mathbf{Y}_i|\theta_{iq};\boldsymbol{\nu})}{f(\mathbf{Y}_i;\boldsymbol{\nu})}\right].
\end{aligned} \tag{S21}$$

In Equation S21, $\nabla_{\boldsymbol{\nu}} H_1(\mathbf{Y}_i; \boldsymbol{\nu})$ is given by Equation S9, and $\nabla_{\boldsymbol{\nu}}[f(\mathbf{Y}_i|\theta_{iq}; \boldsymbol{\nu})/f(\mathbf{Y}_i; \boldsymbol{\nu})]$ is given by Equation S12.

To compute CTT reliability, we apply the transformation function $\varphi_{\text{Rel}}(\mathbf{x}) = (S - x_1^2)/(x_2 - x_1^2)$ where $\mathbf{x} = (x_1, \ldots, x_{2+Q})^\top$ and $S = \sum_{q=1}^{Q} x_{2+q}^2 w_q$. The gradient of φ_{Rel} is

$$\nabla \varphi_{\text{Rel}}(\mathbf{x}) = \left(\frac{2x_1(S - x_2)}{(x_2 - x_1^2)^2}, \frac{-(S - x_1^2)}{(x_2 - x_1^2)^2}, \frac{2w_1 x_3}{x_2 - x_1^2}, \frac{2w_2 x_4}{x_2 - x_1^2}, \ldots, \frac{2w_Q x_{2+Q}}{x_2 - x_1^2} \right)^\top . \tag{S22}$$

B True Item Parameters

B.1 2PL Model

The true item slope (a_j) and difficulty ($b_j = -c_j/a_j$) parameters used in our simulation study in the main document are presented in Tables S1 to S3 for each test length.

Table S1: Data generating parameters for the test with length 8.

**Item**	a_j	b_j
1	0.898	0.330
2	1.058	-0.820
3	1.359	0.487
4	1.862	0.738
5	0.803	0.576
6	1.848	-0.305
7	1.917	1.512
8	1.491	0.390

Table S2: Data generating parameters for the test with length 16.

**Item**	a_j	b_j
1	0.752	-1.219
2	1.711	1.267
3	1.077	-0.745
4	0.992	-1.131
5	1.403	-0.716
6	1.407	0.253
7	0.687	0.152
8	0.942	-0.308
9	1.366	-0.953
10	1.446	-0.648
11	1.268	1.224
12	1.258	0.200
13	1.301	-0.578
14	1.336	-0.942
15	1.802	-0.204
16	1.745	-1.666

Table S3: Data generating parameters for the test with length 32.

**Item**	a_j	b_j	**Item**	a_j	b_j
1	0.898	-0.016	17	1.576	0.388
2	1.058	0.944	18	1.988	-0.054
3	1.359	0.821	19	1.070	-1.377
4	1.862	0.594	20	1.666	-0.415
5	0.803	0.919	21	1.902	-0.394
6	1.848	0.782	22	0.818	-0.059
7	1.917	0.075	23	1.478	1.100
8	1.491	-1.989	24	0.688	0.763
9	1.444	0.620	25	0.901	-0.165
10	0.593	-0.056	26	1.079	-0.253
11	0.809	-0.156	27	0.520	0.697
12	0.765	-1.471	28	1.074	0.557
13	1.531	-0.478	29	1.805	-0.689
14	1.076	0.418	30	1.011	-0.707
15	1.655	1.359	31	1.223	0.365
16	1.247	-0.103	32	1.399	0.769

B.2 Graded Response Model

The true item slope (a_j) and difficulty ($b_{jk} = -c_{jk}/a_j$) parameters used in our simulation study in the main document are presented in Tables S4 to S6 for each test length.

Table S4: Data generating parameters for the test with length 4.

**Item**	a_j	b_{j1}	b_{j2}	b_{j3}	b_{j4}
1	0.881	-0.439	0.548	1.762	2.686
2	1.457	-0.751	0.236	1.449	2.374
3	1.936	-1.518	-0.531	0.683	1.607
4	1.329	-0.884	0.103	1.317	2.241

Table S5: Data generating parameters for the test with length 8.

**Item**	a_j	b_{j1}	b_{j2}	b_{j3}	b_{j4}
1	0.800	-0.644	0.140	1.108	1.894
2	1.528	-1.801	-1.018	-0.049	0.737
3	1.875	-1.736	-0.952	0.016	0.802
4	0.927	-1.818	-1.034	-0.065	0.720
5	0.657	-1.643	-0.859	0.110	0.895
6	1.552	-1.431	-0.647	0.321	1.107
7	1.292	-0.886	-0.102	0.866	1.652
8	1.712	-1.901	-1.117	-0.148	0.637

Table S6: Data generating parameters for the test with length 16.

**Item**	a_j	b_{j1}	b_{j2}	b_{j3}	b_{j4}
1	0.898	-1.212	-0.088	0.901	1.869
2	1.058	-1.653	-0.529	0.460	1.429
3	1.359	-0.744	0.380	1.369	2.337
4	1.862	-1.305	-0.181	0.808	1.777
5	0.803	-1.811	-0.687	0.302	1.271
6	1.848	-2.607	-1.483	-0.495	0.474
7	1.917	-0.938	0.186	1.175	2.144
8	1.491	-1.522	-0.399	0.590	1.559
9	1.444	-1.508	-0.384	0.605	1.573
10	0.593	-1.028	0.096	1.085	2.053
11	0.809	-1.089	0.035	1.023	1.992
12	0.765	-1.203	-0.079	0.910	1.879
13	1.531	-1.041	0.083	1.072	2.041
14	1.076	-1.109	0.015	1.004	1.973
15	1.655	-1.463	-0.339	0.650	1.619
16	1.247	-2.495	-1.371	-0.382	0.587

C Additional Simulation Results

C.1 Simulation Setup

This section presents supplementary simulation results designed to disentangle the effects of test length and target reliability. For the target reliability level of 0.8, we considered all three test lengths under both the 2PL model and the GRM by varying the range of the slope parameters. For target reliabilities of 0.7 and 0.9, however, only two test lengths were examined: the shorter two for a target of 0.7 and the longer two for a target of 0.9 because achieving a reliability of 0.7 with the longest test length or 0.9 with the shortest test length was not feasible with realistic ranges of slope parameters.

Table S7 summarizes the full simulation conditions considered under the 2PL model, where each entry specifies the range of slope parameters used. The diagonal entries corresponding to $a_j \sim \mathcal{U}[0.5, 2]$ represent the conditions reported in the main document. For the GRM, the same distributions for the slope parameters were used under test lengths of $m = 4, 8$, and 16. Difficulty parameters were generated from the same distributions described in the main text. Under the 2PL model, non-convergence occurred when the sample size was $n = 250$, with the highest rate being 0.042. The non-convergent cases were excluded from the analysis.

Table S7: Full simulation conditions under the 2PL model, with the specified uniform distributions used to generate slope parameters for each combination of test length (m) and target reliability. For the GRM, the same slope distributions were used for $m = 4, 8$, and 16.

	Target Reliability		
	0.7	0.8	0.9
$m = 8$	$\mathcal{U}[0.5,\ 2]$	$\mathcal{U}[1,\ 2.5]$	.
$m = 16$	$\mathcal{U}[0.25,\ 1.75]$	$\mathcal{U}[0.5,\ 2]$	$\mathcal{U}[1,\ 2.5]$
$m = 32$	.	$\mathcal{U}[0.25,\ 1.75]$	$\mathcal{U}[0.5,\ 2]$

C.2 Results

Tables S8 and S9 present the full simulation results for PRMSE under the 2PL model and GRM, respectively. Consistent with the results reported in the main document, the SEs were accurately estimated across all simulated conditions, even with small samples. Slight under-coverage was observed under the smallest sample size condition ($n = 250$) due to the skewed sampling distribution, but the coverage improves gradually as the sample size increases. For a given target reliability level, test length did not have any systematic effect on the results, including the bias in point estimates.

Tables S10 and S11 present the full simulation results for CTT reliability under the 2PL model and the GRM, respectively. The SEs were generally accurate when the test

length was short but tended to be overestimated as the test length increased under small samples. Similarly, point estimates exhibited overestimation under small samples, particularly with longer test conditions. When the target reliability level was held constant, increasing the test length tended to increase bias in both the point estimates and the SEs. However, as noted in the main document, larger bias in these estimates does not necessarily result in poor coverage. This is shown in conditions targeting a reliability level of 0.9, where coverage remained satisfactory despite the slightly inaccurate point estimates and SEs. Most importantly, the overestimation in both point estimates and SEs diminished as the sample size increased, and the empirical coverage rates closely matched the nominal level across all conditions when the sample size reached $n = 1000$.

Table S8: Simulation results for PRMSE under the 2PL model. n: Sample size. Target: Target reliability level. m: Test length. True: Population-level PRMSE obtained using true item parameters and population moment calculations. PRMSE Est.: Mean of the PRMSE estimates across replications. Emp. SD: Empirical standard deviation of the point estimates. SE: Mean of the asymptotic SEs estimated by the derived formula. Coverage: Empirical coverage rate of the 95% CI; values falling outside the Monte Carlo error bounds are displayed in bold. LB: Mean lower bound of the CI. UB: Mean upper bound of the CI.

n	Target	m	True	PRMSE Est.	Emp. SD	SE	Coverage	LB	UB
250	0.7	8	0.700	0.708	0.023	0.024	**0.920**	0.662	0.754
		16	0.701	0.709	0.024	0.023	**0.902**	0.664	0.753
	0.8	8	0.787	0.792	0.017	0.017	**0.923**	0.758	0.825
		16	0.801	0.806	0.015	0.015	**0.916**	0.777	0.836
		32	0.820	0.826	0.013	0.014	**0.924**	0.799	0.852
	0.9	16	0.877	0.879	0.008	0.008	**0.920**	0.864	0.895
		32	0.891	0.893	0.007	0.007	**0.920**	0.879	0.907
500	0.7	8	0.700	0.703	0.017	0.017	0.940	0.669	0.736
		16	0.701	0.705	0.016	0.016	0.940	0.673	0.737
	0.8	8	0.787	0.789	0.012	0.012	0.934	0.765	0.814
		16	0.801	0.804	0.010	0.011	0.936	0.783	0.825
		32	0.820	0.823	0.010	0.010	**0.922**	0.804	0.842
	0.9	16	0.877	0.878	0.006	0.006	0.944	0.867	0.889
		32	0.891	0.892	0.005	0.005	0.946	0.883	0.902
1000	0.7	8	0.700	0.701	0.012	0.012	0.950	0.678	0.725
		16	0.701	0.704	0.012	0.012	0.936	0.681	0.727
	0.8	8	0.787	0.788	0.009	0.009	0.956	0.771	0.805
		16	0.801	0.802	0.008	0.008	0.948	0.787	0.817
		32	0.820	0.822	0.007	0.007	0.940	0.808	0.835
	0.9	16	0.877	0.877	0.004	0.004	0.962	0.869	0.885
		32	0.891	0.892	0.003	0.004	0.960	0.885	0.898

Table S9: Simulation results for PRMSE under the GRM. n: Sample size. Target: Target reliability level. m: Test length. True: Population-level PRMSE obtained using true item parameters and population moment calculations. PRMSE Est.: Mean of the PRMSE estimates across replications. Emp. SD: Empirical standard deviation of the point estimates. SE: Mean of the asymptotic SEs estimated by the derived formula. Coverage: Empirical coverage rate of the 95% CI; values falling outside the Monte Carlo error bounds are displayed in bold. LB: Mean lower bound of the CI. UB: Mean upper bound of the CI.

n	Target	m	True	PRMSE Est.	Emp. SD	SE	Coverage	LB	UB
250	0.7	4	0.697	0.707	0.037	0.035	0.934	0.638	0.776
		8	0.708	0.715	0.028	0.029	**0.928**	0.659	0.771
	0.8	4	0.799	0.804	0.022	0.021	**0.912**	0.763	0.844
		8	0.801	0.804	0.018	0.018	**0.922**	0.770	0.839
		16	0.809	0.813	0.016	0.017	0.934	0.779	0.847
	0.9	8	0.887	0.888	0.009	0.009	0.938	0.869	0.906
		16	0.889	0.891	0.010	0.009	**0.918**	0.872	0.909
500	0.7	4	0.697	0.700	0.025	0.024	0.954	0.652	0.748
		8	0.708	0.710	0.019	0.020	0.956	0.670	0.750
	0.8	4	0.799	0.801	0.015	0.015	0.938	0.772	0.830
		8	0.801	0.804	0.012	0.012	0.932	0.779	0.828
		16	0.809	0.811	0.013	0.012	0.936	0.786	0.835
	0.9	8	0.887	0.887	0.007	0.007	0.948	0.874	0.900
		16	0.889	0.889	0.007	0.007	0.942	0.876	0.903
1000	0.7	4	0.697	0.699	0.017	0.017	0.942	0.666	0.732
		8	0.708	0.710	0.014	0.014	0.954	0.682	0.738
	0.8	4	0.799	0.800	0.010	0.010	0.942	0.779	0.820
		8	0.801	0.801	0.009	0.009	0.954	0.784	0.819
		16	0.809	0.810	0.009	0.009	0.934	0.793	0.827
	0.9	8	0.887	0.887	0.005	0.005	0.944	0.878	0.896
		16	0.889	0.890	0.005	0.005	0.950	0.880	0.899

Table S10: Simulation results for CTT reliability under the 2PL model. n: Sample size. Target: Target reliability level. m: Test length. Rel. True: Population-level CTT reliability obtained using true item parameters and population moment calculations. Rel. Est.: Mean of the CTT reliability estimates across replications. Emp. SD: Empirical standard deviation of the point estimates. SE: Mean of the asymptotic SEs estimated by the derived formula. Coverage: Empirical coverage rate of the 95% CI; values falling outside the Monte Carlo error bounds are displayed in bold. LB: Mean lower bound of the CI. UB: Mean upper bound of the CI.

n	Target	m	Rel. True	Rel. Est.	Emp. SD	SE	Coverage	LB	UB
250	0.7	8	0.717	0.730	0.026	0.027	**0.908**	0.677	0.784
		16	0.717	0.731	0.027	0.027	**0.902**	0.679	0.784
	0.8	8	0.795	0.806	0.019	0.020	**0.900**	0.767	0.844
		16	0.808	0.821	0.018	0.019	**0.920**	0.784	0.859
		32	0.826	0.843	0.018	0.023	**0.928**	0.799	0.888
	0.9	16	0.886	0.896	0.012	0.014	0.952	0.870	0.923
		32	0.896	0.915	0.016	0.020	0.952	0.876	0.953
500	0.7	8	0.717	0.722	0.018	0.019	0.938	0.684	0.759
		16	0.717	0.724	0.018	0.019	0.936	0.687	0.761
	0.8	8	0.795	0.800	0.014	0.014	**0.922**	0.773	0.827
		16	0.808	0.815	0.012	0.013	0.944	0.789	0.841
		32	0.826	0.834	0.012	0.014	0.934	0.807	0.861
	0.9	16	0.886	0.891	0.009	0.009	0.946	0.873	0.908
		32	0.896	0.905	0.009	0.011	0.966	0.884	0.926
1000	0.7	8	0.717	0.719	0.013	0.014	0.948	0.693	0.746
		16	0.717	0.721	0.013	0.013	0.936	0.696	0.747
	0.8	8	0.795	0.798	0.009	0.010	0.954	0.779	0.817
		16	0.808	0.811	0.009	0.009	0.946	0.793	0.829
		32	0.826	0.829	0.008	0.009	0.950	0.812	0.846
	0.9	16	0.886	0.889	0.006	0.006	0.944	0.877	0.901
		32	0.896	0.901	0.006	0.007	0.964	0.887	0.914

Table S11: Simulation results for CTT reliability under the GRM. n: Sample size. Target: Target reliability level. m: Test length. Rel. True: Population-level CTT reliability obtained using true item parameters and population moment calculations. Rel. Est.: Mean of the CTT reliability estimates across replications. Emp. SD: Empirical standard deviation of the point estimates. SE: Mean of the asymptotic SEs estimated by the derived formula. Coverage: Empirical coverage rate of the 95% CI; values falling outside the Monte Carlo error bounds are displayed in bold. LB: Mean lower bound of the CI. UB: Mean upper bound of the CI.

n	Target	m	Rel. True	Rel. Est.	Emp. SD	SE	Coverage	LB	UB
250	0.7	4	0.699	0.717	0.038	0.038	**0.910**	0.643	0.791
		8	0.709	0.726	0.031	0.033	**0.920**	0.661	0.791
	0.8	4	0.801	0.816	0.024	0.025	**0.910**	0.767	0.865
		8	0.804	0.820	0.022	0.024	**0.924**	0.772	0.867
		16	0.810	0.838	0.030	0.036	**0.922**	0.768	0.908
	0.9	8	0.889	0.910	0.019	0.022	0.958	0.868	0.953
		16	0.891	0.929	0.030	0.038	0.938	0.854	1.004
500	0.7	4	0.699	0.706	0.025	0.026	0.946	0.656	0.757
		8	0.709	0.717	0.019	0.023	0.948	0.673	0.761
	0.8	4	0.801	0.808	0.016	0.017	**0.928**	0.775	0.840
		8	0.804	0.813	0.014	0.015	**0.926**	0.783	0.844
		16	0.810	0.824	0.018	0.021	0.936	0.783	0.865
	0.9	8	0.889	0.899	0.011	0.012	0.962	0.875	0.922
		16	0.891	0.909	0.015	0.019	0.946	0.871	0.947
1000	0.7	4	0.699	0.703	0.017	0.018	0.936	0.669	0.738
		8	0.709	0.714	0.014	0.015	0.948	0.684	0.744
	0.8	4	0.801	0.804	0.011	0.011	0.948	0.782	0.826
		8	0.804	0.808	0.010	0.010	0.952	0.787	0.828
		16	0.810	0.818	0.011	0.012	0.952	0.794	0.841
	0.9	8	0.889	0.894	0.007	0.007	0.942	0.880	0.909
		16	0.891	0.900	0.008	0.010	0.962	0.880	0.919

D Normalized CTT Reliability

In the main document, we reported (rare) cases where the CTT reliability estimate exceeded its theoretical upper bound of one. In this section, we present a normalized formula that prevents such occurrences, along with necessary derivations. We also provide simulation results for this normalized version of the reliability coefficient and discuss the main findings.

D.1 Normalized Formula

To simplify the notation, let $\tau(\theta_i;\boldsymbol{\nu})$ be the true score of the observed score of interest, $s(\mathbf{Y}_i;\boldsymbol{\nu}) = \mathbb{E}(\Theta_i|\mathbf{Y}_i;\boldsymbol{\nu})$. That is, define $\tau(\theta_i;\boldsymbol{\nu}) = \mathbb{E}[s(\mathbf{Y}_i;\boldsymbol{\nu})|\theta_i;\boldsymbol{\nu}]$. Then, the numerator of Equation 14 in the main document can be expressed as

$$\mathrm{Var}[\tau(\Theta_i;\boldsymbol{\nu});\boldsymbol{\nu}] = \mathbb{E}[\tau(\Theta_i;\boldsymbol{\nu})^2;\boldsymbol{\nu}] - \mathbb{E}[\tau(\Theta_i;\boldsymbol{\nu});\boldsymbol{\nu}]^2. \tag{S23}$$

The second term on the right-hand side of Equation S23 can be reduced to an expectation with respect to $\mathbf{Y}_i$ by the law of iterated expectations:

$$\mathbb{E}[\tau(\Theta_i;\boldsymbol{\nu});\boldsymbol{\nu}]^2 = \mathbb{E}(\mathbb{E}[s(\mathbf{Y}_i;\boldsymbol{\nu})|\Theta_i;\boldsymbol{\nu}])^2 = \mathbb{E}[s(\mathbf{Y}_i;\boldsymbol{\nu});\boldsymbol{\nu}]^2. \tag{S24}$$

However, the first term on the right-hand side of Equation S23 remains an expectation over the continuous LV Θ_i, requiring an approximation of the integral. We proceed to approximate it numerically by quadrature:

$$\mathbb{E}[\tau(\Theta_i;\boldsymbol{\nu})^2;\boldsymbol{\nu}] = \int \tau(\theta_i;\boldsymbol{\nu})^2\phi(\theta_i)d\theta_i \approx \sum_{q=1}^{Q} \tau(\theta_{iq};\boldsymbol{\nu})^2 w_q, \tag{S25}$$

in which $w_q = [\sum_{q=1}^{Q} \phi(\theta_{iq})]^{-1}\phi(\theta_{iq})$, $q = 1,\ldots,Q$, are normalized rectangular quadrature weights. To make Equation S25 estimable using sample data, we further re-express the conditional expectation $\tau(\theta_i;\boldsymbol{\nu})$ as an expectation with respect to the marginal distribution of $\mathbf{Y}_i$:

$$\tau(\theta_i;\boldsymbol{\nu}) = \sum_{\mathbf{y}_i} s(\mathbf{y}_i;\boldsymbol{\nu}) f(\mathbf{y}_i|\theta_i;\boldsymbol{\nu}) = \mathbb{E}\left[s(\mathbf{Y}_i;\boldsymbol{\nu})\frac{f(\mathbf{Y}_i|\theta_i;\boldsymbol{\nu})}{f(\mathbf{Y}_i;\boldsymbol{\nu})}\right], \tag{S26}$$

in which the last equality follows from Bayes' rule. Substituting Equation S26 into the right-hand side of Equation S25 gives

$$\mathbb{E}[\tau(\Theta_i;\boldsymbol{\nu})^2;\boldsymbol{\nu}] \approx \sum_{q=1}^{Q}\left(\mathbb{E}\left[s(\mathbf{Y}_i;\boldsymbol{\nu})\frac{f(\mathbf{Y}_i|\theta_{iq};\boldsymbol{\nu})}{f(\mathbf{Y}_i;\boldsymbol{\nu})}\right]\right)^2 w_q. \tag{S27}$$

The denominator of Equation 14 is first decomposed into two components by the law of total variance:

$$\mathrm{Var}[s(\mathbf{Y}_i;\boldsymbol{\nu})] = \mathrm{Var}[\mathbb{E}(s(\mathbf{Y}_i;\boldsymbol{\nu})|\Theta_i;\boldsymbol{\nu});\boldsymbol{\nu}] + \mathbb{E}[\mathrm{Var}(s(\mathbf{Y}_i;\boldsymbol{\nu})|\Theta_i;\boldsymbol{\nu});\boldsymbol{\nu}]. \tag{S28}$$

The first term on the right-hand side coincides with the numerator shown in Equation S23. To simplify the notation, let $\delta(\theta_i;\boldsymbol{\nu})$ be the conditional variance in the second term: $\delta(\theta_i;\boldsymbol{\nu}) = \mathrm{Var}[s(\mathbf{Y}_i;\boldsymbol{\nu})|\theta_i;\boldsymbol{\nu}] = \mathbb{E}\left[(s(\mathbf{Y}_i;\boldsymbol{\nu}) - \tau(\theta_i;\boldsymbol{\nu}))^2|\theta_i;\boldsymbol{\nu}\right]$. Analogous to Equation S26,

$$\delta(\theta_i;\boldsymbol{\nu}) = \sum_{\mathbf{y}_i}(s(\mathbf{y}_i;\boldsymbol{\nu}) - \tau(\theta_i;\boldsymbol{\nu}))^2 f(\mathbf{y}_i|\theta_i;\boldsymbol{\nu}) = \mathbb{E}\left[(s(\mathbf{Y}_i;\boldsymbol{\nu}) - \tau(\theta_i;\boldsymbol{\nu}))^2\frac{f(\mathbf{Y}_i|\theta_i;\boldsymbol{\nu})}{f(\mathbf{Y}_i;\boldsymbol{\nu})}\right], \tag{S29}$$

which can be estimated from data as the sample average. The expectation $\mathbb{E}[\delta(\Theta_i;\boldsymbol{\nu});\boldsymbol{\nu}]$ is then obtained by approximating the integral using numerical quadrature, in the same manner as Equation S25:

$$\mathbb{E}[\delta(\Theta_i;\boldsymbol{\nu});\boldsymbol{\nu}] = \int \delta(\theta_i;\boldsymbol{\nu})\phi(\theta_i)d\theta_i \approx \sum_{q=1}^{Q}\delta(\theta_{iq};\boldsymbol{\nu})w_q. \tag{S30}$$

Now, to apply the general theory to CTT reliability, we identify $\mathbf{H}$ in Equation 17 as

$$\mathbf{H}(\mathbf{Y}_i;\boldsymbol{\nu}) = (H_1(\mathbf{Y}_i;\boldsymbol{\nu}),\dots,H_Q(\mathbf{Y}_i;\boldsymbol{\nu}),H_{Q+1}(\mathbf{Y}_i;\boldsymbol{\nu}),\dots,H_{2Q}(\mathbf{Y}_i;\boldsymbol{\nu}),H_{2Q+1}(\mathbf{Y}_i;\boldsymbol{\nu}))^\top. \tag{S31}$$

In Equation S31, the last (i.e., the $(2Q+1)$th) component is defined as $H_{2Q+1}(\mathbf{Y}_i;\boldsymbol{\nu}) = s(\mathbf{Y}_i;\boldsymbol{\nu}) = \mathbb{E}(\Theta_i|\mathbf{Y}_i;\boldsymbol{\nu})$; the remaining $2Q$ components pertain to the use of quadratures and are expressed by

$$H_q(\mathbf{Y}_i;\boldsymbol{\nu}) = H_{2Q+1}(\mathbf{Y}_i;\boldsymbol{\nu})\frac{f(\mathbf{Y}_i|\theta_{iq};\boldsymbol{\nu})}{f(\mathbf{Y}_i;\boldsymbol{\nu})}, \quad q = 1,\dots,Q, \tag{S32}$$

and

$$H_{Q+q}(\mathbf{Y}_i;\boldsymbol{\nu}) = (H_{2Q+1}(\mathbf{Y}_i;\boldsymbol{\nu}) - \mathbb{E}[H_q(\mathbf{Y}_i;\boldsymbol{\nu})])^2\,\frac{f(\mathbf{Y}_i|\theta_{iq};\boldsymbol{\nu})}{f(\mathbf{Y}_i;\boldsymbol{\nu})}, \quad q = 1,\dots,Q. \tag{S33}$$

Let $\boldsymbol{\eta} = (\eta_1,\dots,\eta_{2Q+1})^\top$ and $\hat{\boldsymbol{\eta}} = (\hat{\eta}_1,\dots,\hat{\eta}_{2Q+1})^\top$ be the population expectation and the sample mean of Equation S31, respectively. Then, by applying a transformation function $\varphi_{\mathrm{Rel}}:\mathbb{R}^{2Q+1}\to\mathbb{R}$ such that $\varphi_{\mathrm{Rel}}(\mathbf{x}) = (\sum_{q=1}^{Q}x_q^2 w_q - x_{2Q+1}^2)/(\sum_{q=1}^{Q}x_q^2 w_q - x_{2Q+1}^2 + \sum_{q=1}^{Q}x_{Q+q}w_q)$ where

$\mathbf{x} = (x_1, \ldots, x_{2Q+1})^\top$, the population CTT reliability in Equation 14 can be re-expressed by

$$\varphi_{\text{Rel}}(\boldsymbol{\eta}(\boldsymbol{\nu})) = \frac{\sum_{q=1}^{Q} \eta_q^2(\boldsymbol{\nu}) w_q - \eta_{2Q+1}^2(\boldsymbol{\nu})}{\sum_{q=1}^{Q} \eta_q^2(\boldsymbol{\nu}) w_q - \eta_{2Q+1}^2(\boldsymbol{\nu}) + \sum_{q=1}^{Q} \eta_{Q+q}(\boldsymbol{\nu}) w_q}. \tag{S34}$$

Here, the numerator and denominator express Equations S23 and S28, respectively. The corresponding CTT reliability estimate can then be obtained by

$$\varphi_{\text{Rel}}(\hat{\boldsymbol{\eta}}(\hat{\boldsymbol{\nu}})) = \frac{\sum_{q=1}^{Q} \hat{\eta}_q^2(\hat{\boldsymbol{\nu}}) w_q - \hat{\eta}_{2Q+1}^2(\hat{\boldsymbol{\nu}})}{\sum_{q=1}^{Q} \hat{\eta}_q^2(\hat{\boldsymbol{\nu}}) w_q - \hat{\eta}_{2Q+1}^2(\hat{\boldsymbol{\nu}}) + \sum_{q=1}^{Q} \hat{\eta}_{Q+q}(\hat{\boldsymbol{\nu}}) w_q}. \tag{S35}$$

Equation S35 is bounded from above by one because the summation in the denominator is non-negative.

D.2 Additional Derivations

The additional derivations needed are the gradients for the newly defined H functions in Equation S33, which is expressed by

$$\begin{aligned}
\nabla_{\boldsymbol{\nu}} H_{Q+q}(\mathbf{Y}_i; \boldsymbol{\nu}) &= \nabla_{\boldsymbol{\nu}} \left[\left(H_{2Q+1}(\mathbf{Y}_i; \boldsymbol{\nu}) - \mathbb{E}[H_q(\mathbf{Y}_i; \boldsymbol{\nu})]\right)^2 \frac{f(\mathbf{Y}_i | \theta_{iq}; \boldsymbol{\nu})}{f(\mathbf{Y}_i; \boldsymbol{\nu})} \right] \\
&= \nabla_{\boldsymbol{\nu}} \left[\left(H_{2Q+1}(\mathbf{Y}_i; \boldsymbol{\nu}) - \mathbb{E}[H_q(\mathbf{Y}_i; \boldsymbol{\nu})]\right)^2 \right] \frac{f(\mathbf{Y}_i | \theta_{iq}; \boldsymbol{\nu})}{f(\mathbf{Y}_i; \boldsymbol{\nu})} \\
&\quad + \left(H_{2Q+1}(\mathbf{Y}_i; \boldsymbol{\nu}) - \mathbb{E}[H_q(\mathbf{Y}_i; \boldsymbol{\nu})]\right)^2 \nabla_{\boldsymbol{\nu}} \left[\frac{f(\mathbf{Y}_i | \theta_{iq}; \boldsymbol{\nu})}{f(\mathbf{Y}_i; \boldsymbol{\nu})} \right].
\end{aligned} \tag{S36}$$

The first Jacobian term on the second line of Equation S36 is further decomposed as

$$\begin{aligned}
&\nabla_{\boldsymbol{\nu}} \left[\left(H_{2Q+1}(\mathbf{Y}_i; \boldsymbol{\nu}) - \mathbb{E}[H_q(\mathbf{Y}_i; \boldsymbol{\nu})]\right)^2 \right] \\
=&2 \left(H_{2Q+1}(\mathbf{Y}_i; \boldsymbol{\nu}) - \mathbb{E}[H_q(\mathbf{Y}_i; \boldsymbol{\nu})]\right) \left(\nabla_{\boldsymbol{\nu}} H_{2Q+1}(\mathbf{Y}_i; \boldsymbol{\nu}) - \mathbb{E}[\nabla_{\boldsymbol{\nu}} H_q(\mathbf{Y}_i; \boldsymbol{\nu})]\right).
\end{aligned} \tag{S37}$$

In equation S37, $\nabla_{\boldsymbol{\nu}} H_{2Q+1}(\mathbf{Y}_i; \boldsymbol{\nu})$ is given by Equation S9 and $\nabla_{\boldsymbol{\nu}} H_q(\mathbf{Y}_i; \boldsymbol{\nu})$ is given by Equation S21.

To compute CTT reliability, we apply the transformation function $\varphi_{\text{Rel}}(\mathbf{x}) = (S_1 - x_{2Q+1}^2)/(S_1 + S_2 - x_{2Q+1}^2)$ where $\mathbf{x} = (x_1, \ldots, x_{2Q+1})^\top$, $S_1 = \sum_{q=1}^{Q} x_q^2 w_q$, and

$S_2 = \sum_{q=1}^{Q} x_{Q+q} w_q$. The gradient of φ_{Rel} is

$$\nabla\varphi_{\text{Rel}}(\mathbf{x}) = \left(\frac{2x_1 w_1 S_2}{(S_1 + S_2 - x_{2Q+1}^2)^2}, \ldots, \frac{2x_Q w_Q S_2}{(S_1 + S_2 - x_{2Q+1}^2)^2}, \right.$$
$$\left. \frac{-w_1(S_1 - x_{2Q+1}^2)}{(S_1 + S_2 - x_{2Q+1}^2)^2}, \ldots, \frac{-w_Q(S_1 - x_{2Q+1}^2)}{(S_1 + S_2 - x_{2Q+1}^2)^2}, \frac{-2x_{2Q+1} S_2}{(S_1 + S_2 - x_{2Q+1}^2)^2} \right)^\top. \tag{S38}$$

D.3 Simulation Results

The simulation results for the normalized version of the CTT reliability coefficient are presented in Tables S12 and S13, corresponding to the 2PL model and the GRM, respectively. A summary of the main findings is as follows. First, bias in the point estimates and SEs decreased as the sample size increased, consistent with the asymptotic theory. Second, a key distinction from the original formula emerged when the target reliability was high. Specifically, when the reliability approached 0.9, or 0.8 in the case of long tests, the sampling distribution became severely negatively skewed due to the upper bound constraint. This led to underestimation of the point estimates, in contrast to the overestimation observed with the original formula. SEs tended to be accurately estimated under the 2PL model, but under the GRM, SEs were also underestimated when the reliability was high and the test was long. When the sample size reached $n = 1000$, the coverage rates fell within the Monte Carlo error bounds across all conditions.

Table S12: Simulation results for normalized CTT reliability under the 2PL model. n: Sample size. Target: Target reliability level. m: Test length. Rel. True: Population-level CTT reliability obtained using true item parameters and population moment calculations. Rel. Est.: Mean of the CTT reliability estimates across replications. Emp. SD: Empirical standard deviation of the point estimates. SE: Mean of the asymptotic SEs estimated by the derived formula. Coverage: Empirical coverage rate of the 95% CI; values falling outside the Monte Carlo error bounds are displayed in bold. LB: Mean lower bound of the CI. UB: Mean upper bound of the CI.

n	Target	m	Rel. True	Rel. Est.	Emp. SD	SE	Coverage	LB	UB
250	0.7	8	0.717	0.728	0.026	0.027	**0.926**	0.675	0.781
		16	0.717	0.727	0.026	0.026	**0.914**	0.675	0.778
	0.8	8	0.795	0.803	0.018	0.020	**0.925**	0.764	0.841
		16	0.808	0.812	0.016	0.019	0.954	0.774	0.849
		32	0.826	0.822	0.022	0.022	**0.982**	0.779	0.866
	0.9	16	0.886	0.884	0.009	0.013	0.968	0.859	0.909
		32	0.896	0.885	0.018	0.019	**0.992**	0.848	0.922
500	0.7	8	0.717	0.721	0.018	0.019	0.944	0.683	0.758
		16	0.717	0.722	0.018	0.019	0.944	0.685	0.758
	0.8	8	0.795	0.799	0.013	0.014	**0.928**	0.772	0.826
		16	0.808	0.810	0.011	0.013	0.962	0.785	0.835
		32	0.826	0.823	0.013	0.013	0.968	0.797	0.849
	0.9	16	0.886	0.885	0.006	0.008	0.958	0.870	0.900
		32	0.896	0.891	0.008	0.009	**0.986**	0.873	0.910
1000	0.7	8	0.717	0.719	0.013	0.013	0.946	0.693	0.745
		16	0.717	0.720	0.013	0.013	0.940	0.695	0.746
	0.8	8	0.795	0.797	0.009	0.009	0.952	0.779	0.816
		16	0.808	0.809	0.008	0.009	0.956	0.792	0.825
		32	0.826	0.825	0.008	0.008	0.968	0.809	0.840
	0.9	16	0.886	0.885	0.004	0.005	0.970	0.876	0.895
		32	0.896	0.893	0.004	0.005	0.968	0.883	0.904

Table S13: Simulation results for normalized CTT reliability under the GRM. n: Sample size. Target: Target reliability level. m: Test length. Rel. True: Population-level CTT reliability obtained using true item parameters and population moment calculations. Rel. Est.: Mean of the CTT reliability estimates across replications. Emp. SD: Empirical standard deviation of the point estimates. SE: Mean of the asymptotic SEs estimated by the derived formula. Coverage: Empirical coverage rate of the 95% CI; values falling outside the Monte Carlo error bounds are displayed in bold. LB: Mean lower bound of the CI. UB: Mean upper bound of the CI.

n	Target	m	Rel. True	Rel. Est.	Emp. SD	SE	Coverage	LB	UB
250	0.7	4	0.699	0.710	0.037	0.038	0.938	0.637	0.784
		8	0.709	0.715	0.028	0.033	0.950	0.651	0.779
	0.8	4	0.801	0.803	0.022	0.025	0.960	0.753	0.852
		8	0.804	0.803	0.021	0.024	0.958	0.756	0.850
		16	0.810	0.797	0.054	0.034	**0.982**	0.730	0.863
	0.9	8	0.889	0.878	0.018	0.021	**0.994**	0.836	0.919
		16	0.891	0.856	0.061	0.039	**0.992**	0.779	0.934
500	0.7	4	0.699	0.703	0.025	0.026	0.962	0.652	0.753
		8	0.709	0.711	0.019	0.022	0.964	0.667	0.755
	0.8	4	0.801	0.801	0.015	0.017	0.958	0.769	0.834
		8	0.804	0.805	0.013	0.015	0.960	0.776	0.834
		16	0.810	0.801	0.042	0.020	0.968	0.761	0.841
	0.9	8	0.889	0.884	0.010	0.011	**0.980**	0.863	0.905
		16	0.891	0.875	0.026	0.018	**0.978**	0.839	0.911
1000	0.7	4	0.699	0.702	0.017	0.018	0.944	0.667	0.736
		8	0.709	0.711	0.014	0.015	0.958	0.681	0.741
	0.8	4	0.801	0.801	0.011	0.011	0.956	0.779	0.823
		8	0.804	0.804	0.009	0.010	0.966	0.784	0.823
		16	0.810	0.807	0.014	0.012	0.962	0.784	0.830
	0.9	8	0.889	0.887	0.006	0.006	0.964	0.874	0.899
		16	0.891	0.883	0.011	0.009	0.966	0.866	0.901
